# BnaA03.MKK5-BnaA06.MPK3/BnaC03.MPK3 Module Positively Contributes to *Sclerotinia sclerotiorum* Resistance in *Brassica napus*

**DOI:** 10.3390/plants11050609

**Published:** 2022-02-24

**Authors:** Ka Zhang, Chenjian Zhuo, Zhixin Wang, Fei Liu, Jing Wen, Bin Yi, Jinxiong Shen, Chaozhi Ma, Tingdong Fu, Jinxing Tu

**Affiliations:** National Key Laboratory of Crop Genetic Improvement, College of Plant Science and Technology, National Sub-Center of Rapeseed Improvement in Wuhan, Huazhong Agricultural University, Wuhan 430070, China; zhangka@webmail.hzau.edu.cn (K.Z.); zhuocj@webmail.hzau.edu.cn (C.Z.); wangzhixin1989@webmail.hzau.edu.cn (Z.W.); liufeibio@henu.edu.cn (F.L.); wenjing@mail.hzau.edu.cn (J.W.); yibin@mail.hzau.edu.cn (B.Y.); jxshen@mail.hzau.edu.cn (J.S.); yuanbeauty@mail.hzau.edu.cn (C.M.); futing@mail.hzau.edu.cn (T.F.)

**Keywords:** *Brassica napus*, *Sclerotinia sclerotiorum*, mitogen-activated protein kinase, plant defense, molecular mechanism

## Abstract

*Brassica napus* (oilseed rape) is one of the most important oil crops worldwide, but its growth is seriously threatened by *Sclerotinia sclerotiorum*. The mechanism of oilseed rape response to this pathogen has rarely been studied. Here, it was identified that BnaA03.MKK5 whose expression was induced by *S. sclerotiorum* infection was involved in plant immunity. *BnaA03.MKK5* overexpression lines exhibited decreased disease symptoms compared to wild-type plants, accompanied by the increased expression of camalexin-biosynthesis-related genes, including *BnPAD3* and *BnCYP71A13*. In addition, two copies of *BnMPK3* (*BnA06.MPK3* and *BnC03.MPK3*) were induced by *Sclerotinia* incubation, and BnaA03.MKK5 interacted with BnaA06.MPK3/BnaC03.MPK3 in yeast. These interactions were confirmed using in vivo co-immunoprecipitation assays. In vitro phosphorylation assays showed that BnaA06.MPK3 and BnaC03.MPK3 were the direct phosphorylation substrates of BnaA03.MKK5. The transgenic oilseed rape plants including *BnaA06.MPK3* and *BnaC03.MPK3* overexpression lines and *BnMPK3* gene editing lines mediated by CRISPR/Cas9 were generated; the results of the genetic transformation of *BnaA06.MPK3/BnaC03.MPK3* indicate that BnMPK3 also has a positive role in *Sclerotinia* resistance. This study provides information about the potential mechanism of *B. napus* defense against *S. Sclerotiorum* mediated by a detailed BnaA03.MKK5-BnaA06.MPK3/BnaC03.MPK3 module.

## 1. Introduction

*Brassica napus* (genome AACC) is an allotetraploid species derived from the natural hybridization of diploid ancestors *Brassica rapa* (genome AA) and *Brassica oleracea* (genome CC) about 7500 years ago; it contains 19 pairs of chromosomes (A01~A10 and C01~C09) [1]. In nature, oilseed rape is frequently attacked by *Sclerotinia sclerotiorum*, which causes large losses of crop yield [2,3]. *S. sclerotiorum* is a typical necrotrophic fungal pathogen with a wide range of hosts, including many important economic crops such as oilseed rape, peanut, soybean, and sunflower [4].

When challenged by pathogens, the innate immune system of plants is activated. The plant innate immune system has evolved into two branches, pathogen-associated molecular pattern (PAMP)-triggered immunity (PTI) and effector-triggered immunity (ETI) [5,6]. Both PTI and ETI can trigger the activation of kinase cascades mediated by the mitogen-activated protein kinase (MAPK) class [7,8]. The basic MAPK cascade consists of three graded kinases (MAPKKKs (MEKKs), MAPKKs (MKKs) and MAPKs (MPKs)). MPKs are phosphorylated and activated by upstream MPK kinases (MKKs), and MKKs are activated by upstream MKK kinases (MEKKs) through the phosphorylation of conserved Ser/Thr in the activation loop ((S/T)XXXXX(S/T)) of plant MKKs [9]. In addition, MKKs phosphorylate MPKs by acting on Thr/Tyr residues in the activation motif (Thr-X-Tyr) of MPKs, while the common docking (CD) site at the carboxyl-terminal of MPKs includes two adjacent acidic amino acid residues (Asp and Glu), which are essential for the interaction with MKKs [10].

In the *Arabidopsis thaliana* genome, there are 60 MEKKs, 10 MKKs, and 20 MPKs [11]. MEKK1 and MEKK2 are reported to be involved in the salicylic-acid-mediated plant defense response [12]. MKKs can be further divided into four groups (A~D), in which MKK1/2/6 belongs to group A, group B only contains MKK3, MKK4/5 belongs to group C, and group D contains MKK7/8/9/10 [10]. According to the variation of X in the conserved Thr-X-Tyr tripeptide motif, MPKs can be divided into two categories (TEY and TDY), in which TEY can be divided into three groups (A~C), and TDY is classified into group D [9]. The well-studied members MPK3/6 belongs to group A, and MPK4 belongs to group B. MKK1/2 acts upstream of MPK4, and the MKK1/2-MPK4 module works in plant defense against pathogens and tolerance to low temperature and salt stress [12,13]. MKK3 participates in jasmonic acid signal transduction by acting on MPK6 [14]. The two kinases MKK4/5 in group C are involved in plant innate immunity [15,16] and are also related to the development of stomatal and floral organs by phosphorylating MPK3/6 [17,18]. The MKK9-MPK6 module promotes leaf senescence [19].

Many attempts have been made to understand the mechanism of oilseed rape response to *Sclerotinia* infection. Oxalic acid secreted by *S. sclerotiorum* is considered to be one of the main causes of host disease [20]; Liu et al. found that the heterologous overexpression of barley oxalate oxidase coding the BOXO gene in oilseed rape can partially enhance *Sclerotinia* resistance by reducing the oxalate level and increasing the hydrogen peroxide level in transgenic lines [21]. Polygalacturonase secreted by *S. sclerotiorum* is another pathogenic factor [22]; overexpression of the gene encoding rice polygalacturonase inhibitor protein 2 (OsPGIP2) in oilseed rape can improve the stem resistance of *B. napus* to *S. sclerotiorum* [23]. BnWRKY33 is a positive regulatory factor in *Sclerotinia* response. *BnWRKY33*-overexpression lines show less damage than wild-type (WT) plants [24,25]. Wang et al. examined the role of BnMPK3, BnMPK4, and BnMPK6 in *Sclerotinia* resistance and found that the overexpression of these genes could enhance *Sclerotinia* resistance in transgenic lines [26,27,28]. Camalexin, the major phytoalexin in *Arabidopsis* and *Brassicaceae* species, is closely related to defense against *Sclerotinia sclerotiorum* and *Botrytis cinerea* [29,30]. When plants are infected by these pathogens, camalexin-biosynthesis-related genes are induced, and phytoalexins are accumulated. The high expression of camalexin biosynthetic genes contributes to strong resistance to fungal pathogens. Two cytochrome P450 enzymes coding genes, *CYP71A13* and *CYP71B15 (PAD3)*, are involved in camalexin biosynthesis [31].

*S. sclerotiorum* causes great damage to the growth of *B. napus*, but our knowledge about the immune mechanism of oilseed rape against this pathogen is limited; in particular, the role of the MAPK cascade involved in is still obscure. In this study, we provide direct evidence that BnaA03.MKK5 and BnaA06.MPK3/BnaC03.MPK3 act as a module and positively contribute to *Sclerotinia* resistance.

## 2. Results

### 2.1. BnaA03.MKK5 Participated in Response to S. sclerotiorum Infection

**Figure 1:**
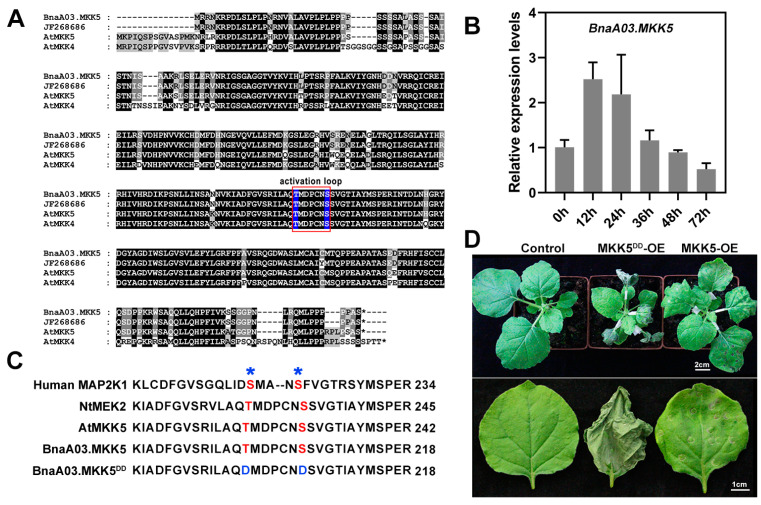
*BnaA03.MKK5* is involved in defense response. (**A**) Sequence alignment of BnaA03.MKK5, JF268686, AtMKK5, and AtMKK4. The conserved amino acid residues are shown in black shading and similar residues are displayed in gray shading. The key motif activation loop is marked with red square frame, and conserved Ser/Thr is highlighted in blue. At, *Arabidopsis thaliana*; Bna, *Brassica napus*. (**B**) Induced expression profiles of *BnaA03.MKK5* were identified at 0 h, 12 h, 24 h, 36 h, 48 h, and 72 h after inoculation with *S. sclerotiorum*. Data are shown as means ± SD (*n* = 3). (**C**) Diagram of the conserved Ser/Thr residues in the activation loop of BnaA03.MKK5. The target sites were detected via alignment to the homologous sequences in other species. BnaA03.MKK5^DD^, conserved Ser/Thr mutated to Asp. Nt, *Nicotiana benthamiana*. (**D**) Constitutively active BnaA03.MKK5^DD^ induced hypersensitive response cell death. Hypersensitive response cell death induced by overexpression of constitutively active BnaA03.MKK5^DD^ (MKK5^DD^-OE) in *N. benthamiana* leaves. MKK5-OE, plants infiltrated with *Agrobacterium* carrying *BnaA03.MKK5* overexpression driven by CaMV35S; MKK5^DD^-OE, plants infiltrated with *Agrobacterium* carrying *BnaA03.MKK5^DD^* overexpression driven by CaMV35S; control, without injection. Scale bars, 2 cm (top image), 1 cm (bottom image).

*B. napus* and model plant *A. thaliana* are Cruciferae members [32]; *S. sclerotiorum* and *B. cinerea*, two closely related necrotrophic fungal pathogens, have large-scale genomic collinearity, share more than 80% amino acid identity, and are similar in terms of their pathogenic mechanisms and metabolites [22]. In order to explore the response mechanism of oilseed rape to *S. sclerotiorum* infection, we focused on the MAPK cascade, including AtMKK5, the role of which in response to biotic and abiotic stress, especially in defense against *B. cinerea* in *A. thaliana,* has been well documented [15]. *AtMKK4* and *AtMKK5* are similar in sequence and functionally redundant [11]. We identified four MKK4 copies and four MKK5 copies in the *B. napus* genome via homologous alignment with AtMKK4 and AtMKK5, respectively, and found that these BnMKK4 and BnMKK5 copies were highly similar in sequence (Figure S1), suggesting that they may have redundant potential functions. Nevertheless, among these BnMKK4/BnMKK5 copies, a paralog named BnMKK4 (GenBank accession No. JF268686) was reported to be involved in low-temperature and salt stress response in *B. napus* [33]. In fact, JF268686 is more similar to AtMKK5 (it shows an identity of 85% with AtMKK5 and 71% with AtMKK4 at the amino acid level), and we cloned this gene in oilseed rape (named *BnaA03.MKK5*) according to the JF268686 sequence (Figure 1A). BnaA03.MKK5 contains a large amount of conserved motifs compared with JF268686 (99.7% identity), AtMKK5 (86% identity) and AtMKK4 (72% identity) (Figure 1A), and *BnaA03.MKK5* could be rapidly induced by *S. Sclerotiorum* (Figure 1B). The transcript abundance of *BnaA03.MKK5* accumulated 12 h after inoculation with *Sclerotinia*, remained at a high level 24 h after infection, and then decreased. This result suggests that *BnaA03.MKK5* may be involved in the early response to *S. sclerotiorum* in oilseed rape. To confirm the role of *BnaA03.MKK5* in defense, we generated an active mutant of BnaA03.MKK5 (named BnaA03.MKK5^DD^), by mutating the conserved Ser/Thr in the activation loop to Asp (Figure 1C). Then, we expressed BnaA03.MKK5 and BnaA03.MKK5^DD^ driven by CaMV35S (cauliflower mosaic virus 35S promoter) in *Nicotiana benthamiana* leaves. It was found that constitutively activated BnaA03.MKK5^DD^, but not BnaA03.MKK5, induced hypersensitive response cell death (Figure 1D). Hypersensitive response cell death is frequently associated with plant disease resistance [16,34]. These data indicate that *BnaA03.MKK5* responds to *S. sclerotiorum* infection and may have a role when plants are challenged by this pathogen.

### 2.2. BnaA03.MKK5 Overexpression Lines Showed Enhanced Resistance to S. sclerotiorum in B. napus

**Figure 2:**
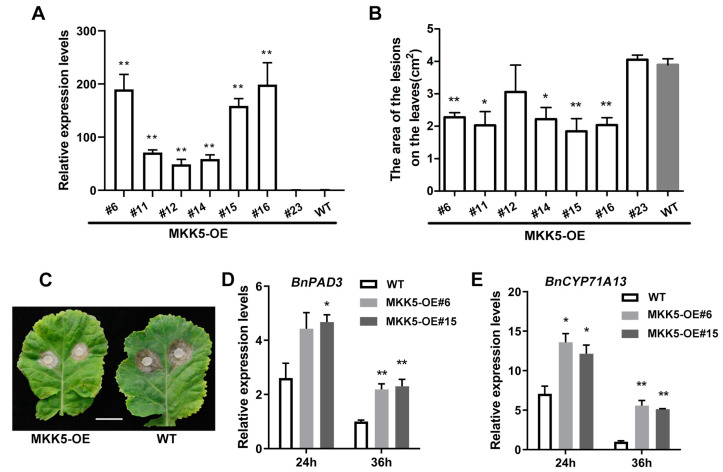
Overexpression of *BnaA03.MKK5* enhances *S. sclerotiorum* resistance in *B. napus.* (**A**) qRT-PCR analysis of *BnaA03.MKK5* overexpression (MKK5-OE) lines. The expression in wild-type (WT) plants is used as the control. (**B**,**C**) Statistical analysis of lesion areas of MKK5-OE lines after 48 h of inoculation, and lesion imaging of representative lines. Scale bar, 2 cm. (**D**,**E**) Relative expression levels of *BnPAD3* and *BnCYP71A13* in MKK5-OE lines and WT plants after inoculation for 24 h and 36 h. In all charts, data are shown as means ± SD (*n* = 3). Asterisks indicate significant differences compared with WT plants at corresponding time points (*t*-test, * *p* < 0.05, ** *p* < 0.01).

To further explore the biological function of BnaA03.MKK5 in defense against *Sclerotinia* in oilseed rape, we generated *BnaA03.MKK5* overexpression (MKK5-OE) transgenic lines. The full-length coding sequence (CDS) of BnaA03.MKK5 driven by CaMV35S was introduced into oilseed rape plants, and seven independent transgenic lines with different expression levels were selected for analysis based on qRT-PCR analysis (Figure 2A). Leaves from approximately 6-week-old T_2_ generation MKK5-OE lines and WT plants were used for inoculation with *S. sclerotiorum*. We measured the lesion area of these infected leaves after 48 h of inoculation (Table S1). The overexpression lines with high expression of *BnaA03.MKK5* tended to show milder lesions than the untransformed WT plants after 48 h of inoculation with *S. Sclerotiorum* (Figure 2B,C), indicating that MKK5-OE lines delay lesion expansion compared with WT plants. We also analyzed the pathogen-induced expression of camalexin biosynthetic genes, including *BnPAD3* and *BnCYP71A13* in two typical MKK5-OE lines after inoculation with *S. Sclerotiorum* for 24 h and 36 h. In contrast to WT plants, the MKK5-OE lines showed increased transcript accumulation of *BnPAD3* and *BnCYP71A13* (Figure 2D,E). These results show that in oilseed rape, the overexpression of *BnaA03.MKK5* significantly enhances plants’ ability to resist the invasion of pathogens when being attacked by *S. sclerotiorum*.

### 2.3. Sequence Analysis of MPK3 in B. napus

Plant MAPK-cascade-mediated signaling is an essential event that occurs in response to pathogens. MKKs function by phosphorylating and activating their downstream MPKs [9,11]. Among the studies concerning the MAPK cascade, most of the elucidated MAPK signaling pathways are related to MAPK3/6 [11]. MAPK3/6 can be activated by multiple upstream kinases such as MKK3/4/5/9, and then are involved in developmental processes, biotic stress responses, and abiotic stress responses. In *A. thaliana*, the MAPK cascade signaling pathway mediated by MKK4/MKK5-MAPK3/MAPK6 module has been reported to play an important role in plant immunity [15,35,36].

**Figure 3:**
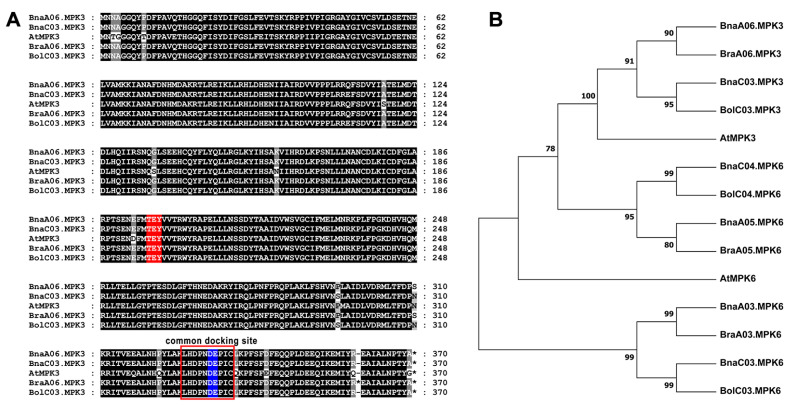
Sequence alignment and phylogenetic tree construction of *Brassica* MPK3. (**A**) Amino acid sequence alignments of identified MPK3 copies in *Brassica* and *Arabidopsis.* The conserved amino acid residues are shown in black shading and similar residues are displayed in gray shading. TEY motif is marked with red; common docking site is marked with red square frame, and two adjacent acidic residues are highlighted in blue. Bra, *Brassica rapa*; Bol, *Brassica oleracea.* (**B**) Phylogenetic analysis showing the relationship between each copy of *B. napus* MPK3 and MPK3/MPK6 in *Brassica* species, as well as MPK3/MPK6 in *Arabidopsis*.

To explore the mechanism of BnaA03.MKK5 in *Sclerotinia* resistance, we considered BnaMPK3 as its potential substrate. Through sequence alignment and the homology cloning approach, we identified two orthologs of AtMPK3 in oilseed rape, which were located on chromosomes A06 and C03, respectively, namely BnaA06.MPK3 and BnaC03.MPK3. There was only one ortholog of AtMPK3 in *B. rapa*, named BraA06.MPK3; only one ortholog of AtMPK3 was identified in *B. oleracea*, named BolC03.MPK3. Sequence analysis of MPK3 in *B. napus*, *B. rapa*, *B. oleracea,* and *A. thaliana* showed that the amino acid sequences of these MPK3 members were almost identical (they shared over 94% identity). These *Brassica* MPK3, like AtMPK3, contained conserved TEY domains and a common docking site (Figure 3A). Phylogenetic analyses were conducted to investigate the evolutionary relationship of BnaA06.MPK3/BnaC03.MPK3 to MPK3 members of other representative species (Figure 3B). BnaA06.MPK3 was more closely related to BraA06.MPK3, and BnaC03.MPK3 was more closely related to BolC03.MPK3. That is, BnaA06.MPK3 inherited from the copy in *B. rapa*, and BnaC03.MPK3 inherited from the copy in *B. oleracea*. BnaA06.MPK3/BnaC03. MPK3 showed a closer genetic relationship with AtMPK3 and AtMPK6, indicating that BnaA06.MPK3/BnaC03.MPK3 may have similar biological functions with these two kinases (Figure 3B).

### 2.4. BnaA06.MPK3 and BnaC03.MPK3 Are the Phosphorylation Substrate of BnaA03.MKK5

**Figure 4:**
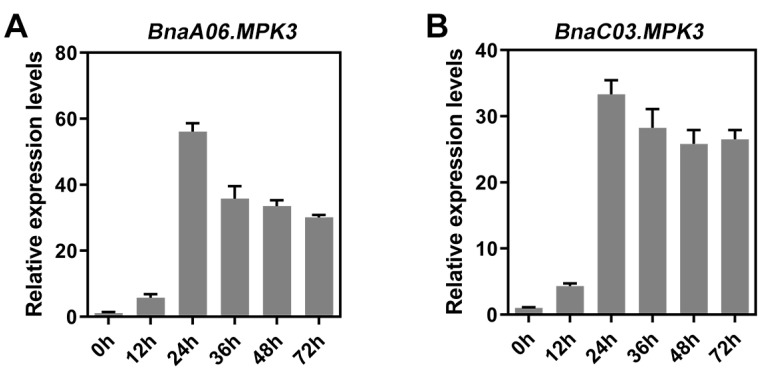
Expression analysis of two BnMPK3 copies in response to *Sclerotinia* infection. Induced expression profiles of *BnaA06.MPK3* (**A**) and *BnaC03.MPK3* (**B**) were identified at 0 h, 12 h, 24 h, 36 h, 48 h, and 72 h after inoculation with *S. sclerotiorum*. Data are shown as means ± SD (*n* = 3).

qRT-PCR was performed to determine the expression profiles of *BnaA06.MPK3* and *BnaC03.MPK3* in WT oilseed rape leaves infected with *S. sclerotiorum* within 72 h. *BnaA06.MPK3* and *BnaC03.MPK3* could be significantly induced by *S. Sclerotiorum*; the expression levels peaked at 24 h after inoculation and then decreased, but the levels still remained high (Figure 4A,B). These data suggest that BnaA06.MPK3 and BnaC03.MPK3, similar to BnaA03.MKK5, are involved in the defense mechanism against *S. sclerotiorum*.

**Figure 5:**
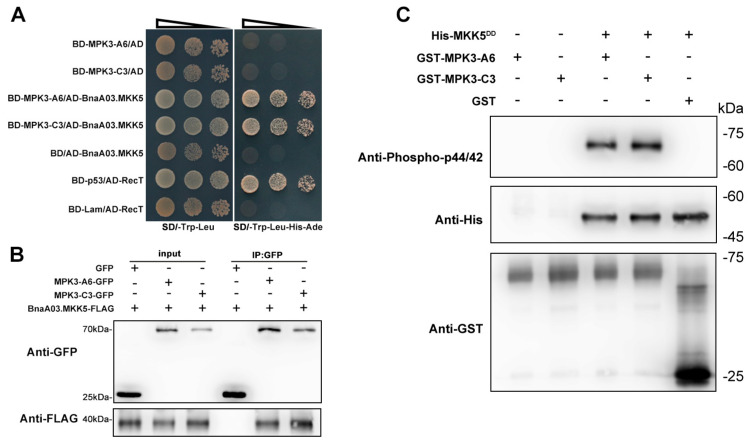
Identification of the interaction between BnaA03.MKK5 and BnaA06.MPK3/BnaC03.MPK3. (**A**) Yeast two-hybrid (Y2H) to detect the interaction between BnaA03.MKK5 and BnaA06.MPK3/BnaC03.MPK3. BD-p53/AD-RecT, positive control; BD-Lam/AD-RecT, negative control. (**B**) Co-immunoprecipitation (CoIP) assays to confirm the interaction between BnaA03.MKK5 and BnaA06.MPK3/BnaC03.MPK3 in planta. (**C**) In vitro phosphorylation detection of BnaA03.MKK5 on BnaA06.MPK3/BnaC03.MPK3.

To determine whether BnaA06.MPK3/BnaC03.MPK3 and BnaA03.MKK5 work as a module to participate in *Sclerotinia* resistance, we first conducted assays to examine the interaction between these kinases. In Y2H assays, the full-length CDS of *BnaA03.MKK5* was inserted into the pGADT7 vector, and *BnaA06.MPK3/BnaC03.MPK3* was cloned into the pGBKT7 vector. Yeast cells transformed with the combination of a pGBKT7 empty vector, and AD-MKK5 (BD/AD-MKK5) grew normally on selective dropout medium SD/-Trp Leu, but not on SD/-Trp Leu His Ade. However, when replacing the pGBKT7 empty vector with BD-MAPK3-A6 or BD-MAPK3-C3, the yeast cells could grow normally, even on selective dropout medium SD/-Trp Leu His Ade. In addition, the combination of BD-MAPK3-A6/AD and BD-MAPK3-C3/AD could not grow on SD/-Trp Leu His Ade, indicating that BnaA06.MAPK3/BnaC03.MAPK3 has no self-activation in the Y2H system (Figure 5A). These results suggest that BnA03.MKK5 interacts with BnaA06.MAPK3/BnaC03.MAPK3. The interactions were further confirmed using co-immunoprecipitation (CoIP) assays, in which BnaA03.MKK5-FLAG, BnaA06.MPK3-GFP and BnaC03.MPK3-GFP fusion proteins were coexpressed in *N. benthamiana* leaves (Figure 5B). BnaA03.MKK5-FLAG co-immunoprecipitated with BnaA06.MPK3-GFP or BnaC03.MPK3-GFP instead of the negative control, indicating that BnaA03.MKK5 can form a protein complex with BnaA06.MAPK3/BnaC03.MAPK3 in vivo. Next, in vitro phosphorylation assays were performed to determine whether BnaA06.MAPK3/BnaC03.MAPK3 could be phosphorylated directly by upstream BnaA03.MKK5. We expressed GST-tagged BnaA06.MPK3/BnaC03.MPK3 fusion protein in *E. coli* and purified the protein samples with GST beads. The phosphorylation signal was only detected when BnaA06.MPK3/BnaC03.MPK3 was incubated with BnaA03.MKK5^DD^ (Figure 5C). Taken together, the in vitro and in vivo results indicate that BnaA03.MKK5 physically interacts with and phosphorylates BnaA06.MPK3 or BnaC03.MPK3.

### 2.5. BnaA06.MPK3/BnaC03.MPK3 Positively Contribute to S. sclerotiorum Resistance in B. napus

**Figure 6:**
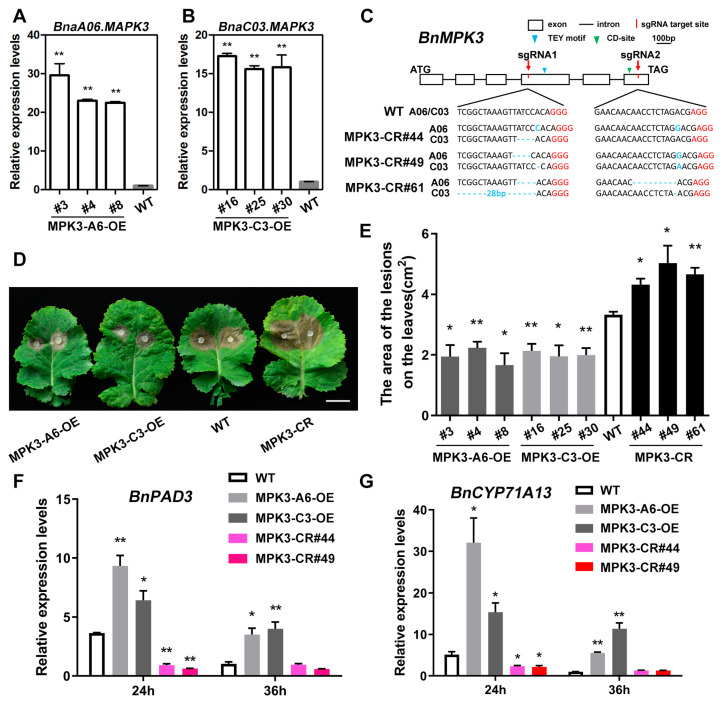
Contribution of *BnMPK3* to *S. sclerotiorum* resistance in *B. napus.* (**A**,**B**) *BnaA06.MPK3* overexpression (MPK3-A6-OE) lines (**A**) and *BnaC03.MPK3* overexpression (MPK3-C3-OE) lines (**B**) established using qRT-PCR analysis. The expression in WT plants as control. Data are shown as means ± SD (*n* = 3). Asterisks indicate significant differences compared with WT plants (*t*-test, ** *p* < 0.01). (**C**) A schematic diagram to show the characterization of the *BnMPK3* exon/intron sequence and the target sites of sgRNAs. The edit types of three independent homozygous mutants (MPK3-CR#44, 49, and 61) are shown. The protospacer-adjacent motifs (PAM) are marked in red. TEY motif, phosphorylated by MKKs; CD-site, interacts with MKKs; ATG, start codon; TAG, stop codon. (**D**) Photographs of the lesions of detached leaves from *BnMPK3* transgenic lines and WT plants in approximately 4 weeks when inoculated with 7 mm *S. sclerotiorum* hyphae agar block at 22 °C for 48 h. Scale bar, 2 cm. (**E**) Statistical analysis of the lesion areas of *BnMPK3* transgenic lines and WT plants after 48 h inoculation. Data are shown as means ± SD (*n* = 3). Asterisks indicate significant differences compared with WT (*t*-test, * *p* < 0.05, ** *p* < 0.01). (**F**,**G**) Relative expression levels of two camalexin biosynthesis-related genes, *BnPAD3* and *BnCYP71A13*, in *BnMPK3* transgenic lines and WT plants after *S. sclerotiorum* inoculation for 24 h or 36 h. Data are shown as means ± SD (*n* = 3). Asterisks indicate significant differences compared with WT at corresponding time points (*t*-test, * *p* < 0.05, ** *p* < 0.01).

We generated transgenic oilseed rape plants with *BnaA06.MPK3-* and *BnaC03.MPK3-* overexpression driven by CaMV35S, *BnaMPK3* knockout mediated by CRISPR/Cas9. Three independent BnaA06.MPK3 overexpression (MPK3-A6-OE) lines and three independent BnaC03.MPK3 overexpression (MPK3-C3-OE) lines with more than 10-fold increases in expression were used for further analysis (Figure 6A,B). In addition, three independent homozygous mutants with different editing types in the genome region of *BnaA06.MPK3* and *BnaC03.MPK3* were established for comparison with WT plants (Figure 6C). The conserved amino acid motif TDY/TEY of MAPKs is phosphorylated by MKKs, and the common docking (CD) site located in the carboxyl-terminal of MAPKs includes two adjacent acidic residues (D and E) that are crucial for the interaction with MKKs [10]. The insertions/deletions mediated by CRISPR/Cas9 lead to out-of-frame mutation and the destruction of key domains of target genes. CR#44 carried a 1-bp insertion in sgRNA1 of A06, a 1-bp insertion in sgRNA2 of A06, and a 4-bp deletion in sgRNA1 of C03; CR#49 carried a 4-bp deletion in sgRNA1 of A06, a 1-bp insertion in sgRNA2 of A06, a 1-bp deletion in sgRNA1 of C03, and a 1-bp insertion in sgRNA2 of C03. CR#61 carried a 4-bp deletion in sgRNA1 of A06, a 9-bp deletion in sgRNA2 of A06, a 28-bp deletion in sgRNA1 of C03, and a 1-bp deletion in sgRNA2 of C03. After 48 h of inoculation with *S. sclerotiorum*, it was determined that the lesion areas of both *BnaA06.MPK3* and *BnaC03.MPK3* overexpression lines were smaller than those in WT plants. However, the knockout lines showed a decrease in pathogen resistance (Figure 6D,E and Table S1). The expression of *BnPAD3* and *BnCYP71A13* in *BnaA06.MPK3* and *BnaC03.MPK3* overexpression lines were much higher than those in WT plants and remained at a high level with the invasion of pathogens. In *BnMPK3* knockout lines, the expressions of these genes did not change much and were at a very low level (Figure 6F,G). These results show that in *B. napus*, BnaA06.MPK3/BnaC03.MPK3 has a positive role in inhibiting the damage of pathogens to plants.

## 3. Discussion

*S. sclerotiorum* is one of the most harmful diseases to oilseed rape growth. Great efforts have been made to identify the quantitative genetic loci (QTL) defense against *Sclerotinia* in oilseed rape [37,38,39]. No germplasm in oilseed rape, or even in all *Brassicaceae* species, is immune to *Sclerotinia*, which has made the propagation of resistant varieties less effective for decades, and disease control in oilseed rape primarily relies on partially resistant lines [23,39]. Moreover, little progress has been made with regard to the identification of resistant germplasms and genes in *B. napus*, making it difficult to study the interaction between oilseed rape and *Sclerotinia*, and the potential molecular mechanisms of oilseed rape response to this pathogen are still poorly understood. As there is no resistance (R) gene in oilseed rape, the plant’s innate immune system may be the primary solution to defense against *S. sclerotiorum* for oilseed rape. The MAPK cascade has a significant role in response to pathogens. In this study, we found that the BnaA03.MKK5-BnaA06.MPK3/BnaC03.MPK3 module positively contributes to *S. sclerotiorum* resistance in *B. napus*. Unfortunately, the MKK5-MPK3 cascade is conserved in the stress response, which means this study lacks novelty. However, it is the first work to identify a detailed MAPK cascade module in *Sclerotinia* resistance in oilseed rape, which provides new evidence that could reveal the complex response mechanism of oilseed rape defense against *Sclerotinia*. The results of this study also suggest that the transgenic manipulation of factors associated with the innate immune system provides alternative approaches for the creation of diverse oilseed rape varieties with *Sclerotinia* resistance.

Four copies of MKK5 were identified in the *B. napus* genome, located on chromosomes A01, A03, C01, and C03, respectively, and four copies of MKK4 were found on chromosomes A05, A08, C03, and C06 in the *B. napus* genome. In oilseed rape, these MKK4 and MKK5 copies exhibited sequence identity and contained a typical activation loop at the C-terminus (Figure S1). In addition, we analyzed the sequences of all MKK4/MKK5 copies from *B. napus*, *B. rapa*, *B. oleracea,* and *A. thaliana*. Based on phylogenetic analysis (Figure S2), we found that BnaA01.MKK5 was more closely related to BraA01.MKK5, while BnaC01.MKK5 was closer to BolC04.MKK5; the BnaA01.MKK5 copy was inherited from *B. rapa*, while the BnaC01.MKK5 copy was inherited from *B. oleracea*. Similarly, BnaA03.MKK5 and BnaC03.MKK5 were orthologs of those in the *B. rapa* and *B. oleracea* genome, respectively, and were also parahomologs in the A and C subgenomes of *B. napus*. In general, BnaA01.MKK5, BnaC01.MKK5, BnaA03.MKK5, and BnaC03.MKK5 were orthologs of AtMKK5. In *Arabidopsis*, MKK4 and MKK5 are two closely related MKKs that are functionally interchangeable with NtMEK2 in activating downstream MPKs [16]. The independent roles of AtMKK4 and AtMKK5 have not been revealed. Gene editing mediated by the CRISPR/Cas system has been successful and widely used, especially in targeting functional redundant genes. The abscisic acid (ABA) receptor family (PYL) consists of 14 genes with redundant functions in the model plant *Arabidopsis* [40]. Miao et al. generated *pyl* mutants with seven genes edited in rice using the CRISPR/Cas9 system and identified their effects on growth [41]. With the continuous development of gene editing technology, the detailed function of *B. napus* genes will be further revealed by mutant creation with multiple copies, including BnMKK4 and BnMKK5. In this work, we obtained *bnmpk3* mutants that were edited on two copies and resulted in the deletion of the functional domain.

The *Brassica* species experienced a triploidization event in its evolution history, resulting in genome duplications of *B. rapa* and *B. oleracea* compared with *Arabidopsis* [1,42]. In addition, due to the fact that *B. napus* is a heterologous tetraploid derived from *B. rapa* and *B. oleracea*, in general, there are more than two homologs of one *A. thaliana* gene in the *B. napus* genome. However, MPK3 has only two copies in *B. napus* and one copy in *B. rapa* or *B. oleracea* (Figure 3A), meaning that this gene is alienated in the process of *Brassica* triploidization. Genome duplication is a driving force of plant evolution and is the primary way in which plants adapt to their natural environment [43]. In fact, not all duplicated genes inherited normal biological functions during genome evolution history; most duplicated gene pairs experienced pseudogenization or gene losses, whereas a few duplicate gene pairs were retained [44]. Gene loss is almost always accompanied by genome duplication. In addition, there are two homologs of AtMPK6 in the A or C subgenome, respectively, following the polyploid event of *Brassica*. These *Brassica napus* MPK6 are highly similar with BnaA06.MPK3 and BnaC03.MPK3, sharing conserved sub-domains and key motifs (Figure S3). AtMPK3/AtMPK6 is involved in biotic and abiotic stress, and AtMPK6 is also involved in plant growth and development. AtMPK3 and AtMPK6 are both phosphorylation substrates of AtMKK4/AtMKK5, showing functional redundancy [15,16,17,18]. This contributes to survival stability and prevent complete function loss in the case of gene mutation.

Phosphorylation-activated MAPKs act on downstream substrates, and transcription factors are widely reported to be phosphorylated by MAPK cascade. In *Arabidopsis*, activated MPK3/6 can directly phosphorylate downstream substrates, such as WRKY33, ERF104, and ICE1 [45,46,47]. The constitutively activated MKK4^DD^/MKK5^DD^ is used as an upstream activator of the MAPK cascade in the in vitro phosphorylation assay of MPK3/6 on its substrate [46,47]. In this study, BnaA03.MKK5^DD^ is a proved working activator and can be effectively used in the related research on MAPK cascade in *B. napus*.

In conclusion, we developed a detailed MAPK cascade module mediated by BnaA03.MKK5-BnaA06.MPK3/BnaC03.MPK3 in *B. napus* and proved that this module positively contributed to *S. sclerotiorum* resistance of *B. napus*. All *B. napus* MKK5 and MPK3 copies showed large-scale sequence identity, indicating that there is likely to be functional redundancy among copies or closely related members. Subsequently, it was identified that the BnaA03.MKK5, BnaA06.MPK3, and BnaC03.MPK3 copies were induced by *Sclerotinia* infection. Both BnaA06.MPK3 and BnaC03.MPK3 interacted with BnaA03.MKK5 and were directly phosphorylated by the upstream BnaA03.MKK5. This study not only reveals a detailed MAPK module, but also determines the function of the detailed MAPK cascade copies, laying the foundation for the further research of the response mechanisms against *S. sclerotiorum* in *B. napus*.

## 4. Materials and Methods

### 4.1. Plant Materials, Growth Conditions and Plasmid Construction

The *B. napus* varietie Jia 9709 were used to establish transgenic lines, including *BnaA03.MKK5-OE, BnaA06.MPK3-OE*, *BnaC03.MPK3-OE,* and *BnaMPK3-CR*. Wild-type and transgenic oilseed rape plants were grown in a field at the Wuhan experimental base for seed propagation. For oilseed rape leaf inoculation and *Nicotiana benthamiana* growth, seedlings were grown in a greenhouse (22 °C, 16 h day/8 h night). The full-length coding sequences of *BnaA03.MKK5* (BnaA03g36120D)*, BnaA06.MPK3* (BnaA06g18440D), and *BnaC03.MPK3* (BnaC03g55440D) were driven by CaMV35S to generate overexpression constructs; the *BnaMPK3* gene editing construct mediated by CRISPR/Cas9 was designed as previously described [48], and the sgRNA of targeted genes was designed using CRISPR-P (http://cbi.hzau.edu.cn/cgi-bin/CRISPR, 23 February 2022). The recombinant plasmids were confirmed via sequencing and were introduced into *Agrobacterium tumefaciens* strain GV3101 and transferred to Jia 9709 in accordance with the previously mentioned method [49]. The primers for the constructs are listed in Table S2.

### 4.2. RNA Extraction and qRT-PCR

RNA was isolated from transgenic lines and WT plants (treated with pathogens) using the RNAprep pure plant kit (TIANGEN, DP441, Beijing, China). Then, 1 µg of total RNA was used for cDNA synthesis using the RevertAid™ First Strand cDNA Synthesis Kit (Fermentas, #K1622, Waltham, MA, USA). qRT-PCR analysis was performed with the CFX96^TM^ Real-Time system (Bio-Rad, Berkeley, CA, USA) using the SYBR Green Realtime PCR Master Mix (TOYOBO, QPK-201, Osaka, Japan). *BnACTIN2* was used as a control to normalize expression levels according to the 2^−ΔΔCT^ method. The primers used in the qRT-PCR assays are given in Table S2.

### 4.3. Pathogen Inoculation and Lesion Measurement

*S. sclerotiorum* A367 was used for inoculation and was cultured on potato dextrose agar medium (20% potato, 2% dextrose and 1.5% agar) at 22 °C in the dark. The latest fully unfolded leaves were removed from oilseed rape plants of approximately 6 weeks, and the detached leaves were placed on soaked gauze. Then, the mycelial side of the fungus agar block (7 mm in diameter) was attached to the leaf surface. The inoculated leaves were covered with plastic films to maintain moisture at 22 °C. After 48 h of inoculation, the images of inoculated leaves were taken and the lesion area was measured using ImageJ software.

### 4.4. Sequence Alignment and Phylogenetic Analysis

Sequence analysis was based on *B. napus* reference genome *Darmor-bzh* V5, the BnTIR (http://yanglab.hzau.edu.cn/BnTIR, 23 February 2022) database and The Arabidopsis Information Resource (TAIR, TAIR 10) database. Sequence alignments were conducted using the ClustalW program in MEGA software (MEGA_11.0.10), and the results were displayed using the GENEDOC software (GeneDoc 2.7.0.0) with manual editing. The unrooted phylogenetic tree was constructed using the Maximum Likelihood method with 1000 replicates for bootstrap analysis, and MEGA software was used to display the result.

### 4.5. Yeast Two-Hybrid (Y2H) Assay

The Y2H assays were performed following the manufacturer’s protocol using the Matchmaker GAL4 Two-Hybrid System (Clontech, Shiga, Japan). The full-length *BnaA03.MKK5* CDS was cloned into the pGADT7 vector, and the full-length CDSs of *BnaA06.MPK3* and *BnaC03.MPK3* were inserted into the pGBKT7 vector. To test protein-protein interactions, the recombinant pGADT7 and pGBKT7 constructs were co-transformed into an AH109 yeast strain. Yeast cells were grown on selective dropout medium SD/-Trp Leu (lacking Trp and Leu) and SD/-Trp Leu His Ade (lacking Trp, Leu, His and Ade). The construct primers are listed in Table S2.

### 4.6. In Vivo Co-Immunoprecipitation (CoIP) Assay

The coding sequences of BnaA06.MPK3 and BnaC03.MPK3 were cloned into the *pH7LIC6.0* vector to generate GFP fusion proteins, and the coding sequence of BnaA03.MKK5 was cloned into the *pH7LIC4.1* vector to generate GFP fusion proteins. The recombinant GFP and FLAG constructs in *A. tumefaciens* strain GV3101 were transiently co-expressed in 4-week-old *N. benthamiana* leaves for 3 days. An amount of ~5 g of infiltrated leaves was sampled, and the total protein was extracted using 5 mL of lysis buffer (50 mM Tris-HCl pH 7.5, 150 mM NaCl, 1% Triton X-100, 5 mM EDTA, 10% glycerol, and 1 × protease inhibitor (Roche, Cat. No. 11836153001, Mannheim, Germany). Then, the samples were incubated with 25 μL of GFP-Trap-MA (Chromotek, gtma-20, Munich, Germany) at 4 °C for 1 h, followed by being washed five times with washing buffer (0.1% Triton X-100 instead of 1% Triton X-100 in lysis buffer). The coupled target proteins were eluted with SDS loading buffer and detected with anti-FLAG (Abclonal, AE005, Wuhan, China, 1:10,000) and anti-GFP (Abclonal, AE012, Wuhan, China, 1:10,000) antibodies. The construct primers are listed in Table S2.

### 4.7. In Vitro Phosphorylation Assay

The coding sequences of BnaA06.MPK3 and BnaC03.MPK3 were fused with GST tag (*pGEX4T-1* plasmid was used), and the coding sequence of BnaA03.MKK5^DD^ was cloned into the *pET32a* plasmid (6× His was fused in the N-terminus of MKK5). The recombinant GST-BnaA06.MPK3, GST-BnaC03.MPK3, and His-BnaA03.MKK5^DD^ proteins were expressed in *Escherichia coli* strain BL21. Cells were harvested after 16 h of incubation with 0.2 mM isopropyl-beta-D-thiogalactopyranoside (IPTG) at 16 °C, and then, they were lysed using a high-pressure cell disrupter (JNBIO, Guangzhou, China). The protein samples were purified using GST-Sefinose resin (BBI, C600031, Shanghai, China) and Ni^2+^ affinity resin (BBI, C600033, Shanghai, China). The BnaA03.MKK5-BnaA06.MPK3/BnaC03.MPK3 phosphorylation assay was performed as previously described [50]. GST-tagged BnaA06.MPK3/BnaC03.MPK3 were incubated with His-tagged BnaA03.MKK5^DD^ in the reaction buffer (50 μM ATP, 50 mM HEPES pH 7.5, 10 mM MgCl_2_, and 2 mM dithiothreitol) at 25 °C for 1 h. Phosphorylation signals were detected using anti-Phospho-p44/42 antibody (CST, 4370T, Danvers, MA, USA, 1:2000). The recombinant BnaA03.MKK5^DD^ was detected with anti-His antibody (Abclonal, AE003, Wuhan, China, 1:10,000). The recombinant BnaA06.MPK3 and BnaC03.MPK3 were detected with anti-GST antibody (Abclonal, AE001, Wuhan, China, 1:10,000). The construct primers are listed in Table S2.

## Data Availability

The data that support the findings of this study are available in the Supplementary Materials of this article.

## Supplementary Materials

The following supporting information can be downloaded at: https://www.mdpi.com/article/10.3390/plants11050609/s1. Figure S1: Sequence alignment of identified MPK4/5 copies in *Brassica napus* and *Arabidopsis*. Figure S2: Phylogenetic analysis showing the relationship between each copy of B. napus MKK5 and MKK4/5 in *Brassica* species, as well as MKK4/5 in *Arabidopsis*. Figure S3: Sequence alignment of *B. napus* MPK3/6 copies. Table S1: Lesion areas of infected oilseed rape leaves after 48 h incubation with *S. Sclerotiorum*. Table S2: Primers used in this study.

## Abbreviations

MAPK/MPK	Mitogen-activated protein kinase
MAPKK/MKK/MEK	Mitogen-activated protein kinase kinase (MAPK kinase)
MAPKKK/MEKK	Mitogen-activated protein kinase kinase kinase (MAPKK kinase)
MPK3	Mitogen-activated protein kinase 3
MKK5	Mitogen-activated protein kinase kinase 5
PAMP	Pathogen-associated molecular pattern
PTI	Pathogen-associated molecular pattern (PAMP)-triggered immunity
ETI	Effector-triggered immunity
CD site	Common docking site
WT	Wild-type
Bn/Bna	Brassica napus
At	Arabidopsis thaliana
^DD^	Conserved Ser/Thr were mutated to Asp
CaMV35S	Cauliflower mosaic virus 35S promoter
OE	Overexpression
CDS	Coding sequence
qRT-PCR	Quantitative real-time PCR
PAD3	PHYTOALEXIN DEFICIENT 3
CYP71A13	CYTOCHROME P450, FAMILY 71, SUBFAMILY A, POLYPEPTIDE 13
± SD	± standard deviation
Y2H	Yeast two-hybrid
AD	Activation domain
BD	Binding domain
SD/-Trp Leu	Selective dropout medium lacking Trp and Leu
CoIP	co-immunoprecipitation
GST	Glutathione S-transferase
GFP	Green fluorescent protein
QTL	Quantitative genetic loci
C-terminus	Carboxyl-terminus
CRISPR	Clustered regularly interspaced short palindromic repeats
cDNA	Complementary DNA
