# Supplementary material for: BnaA03.MKK5-BnaA06.MPK3/BnaC03.MPK3 Module Positively Contributes to Sclerotinia sclerotiorum Resistance in Brassica napus
Source: Plants (Basel). 2022 Feb 24;11(5):609. doi: 10.3390/plants11050609 (PMC8912397; doi:10.3390/plants11050609)
Supplement: Supplementary file 1 [file plants-11-00609-s001.zip › Supplementary Figures.pdf]

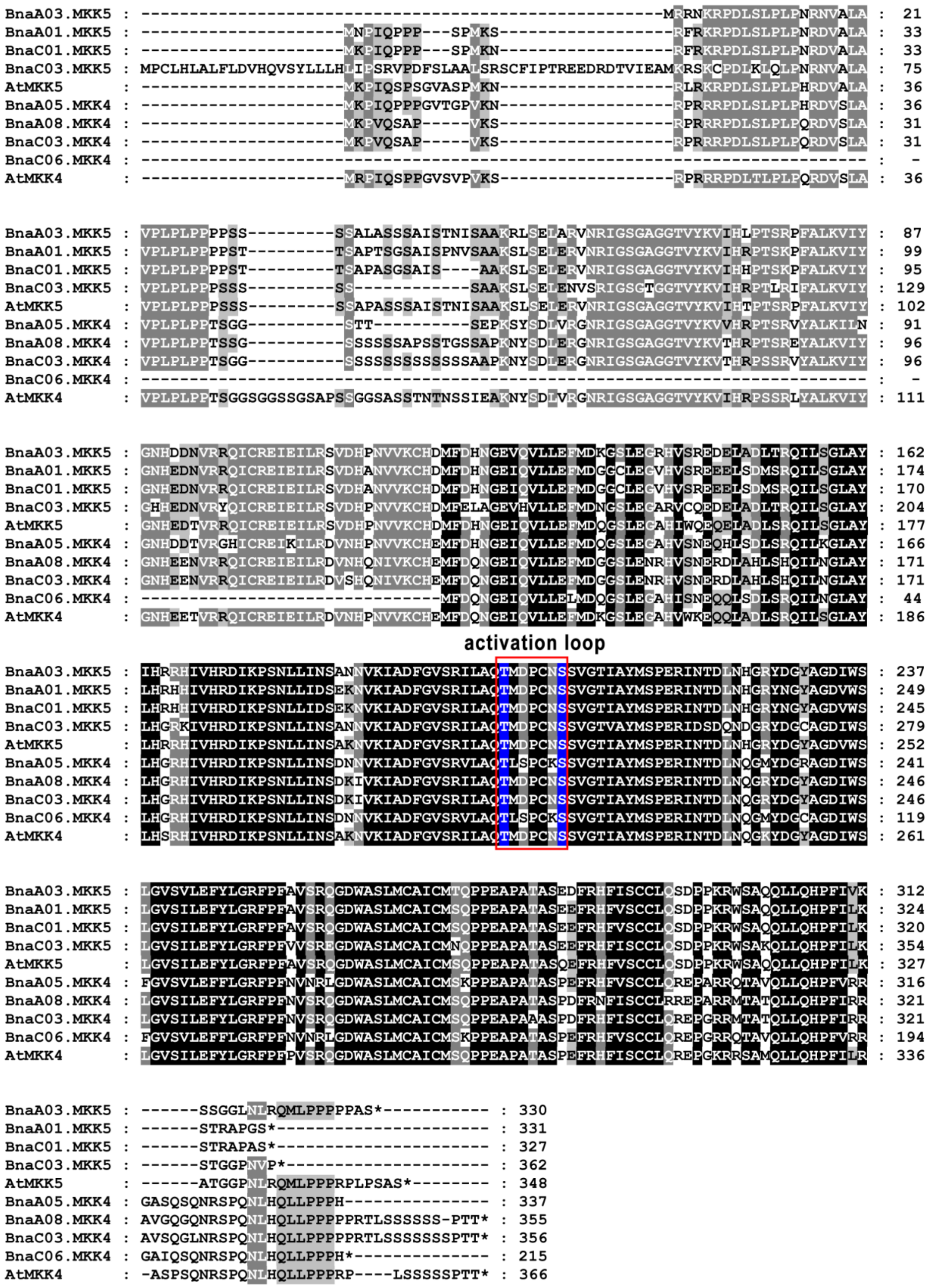

Figure S1: Sequence alignment of identified MPK4/5 copies in *Brassica napus* and *Arabidopsis*. Amino acid sequences were used for alignment, and the identical amino acids and similar amino acids were displayed with black and grey shading respectively. The activation loop was marked with red square frame, and conserved Ser/Thr was highlighted in blue.

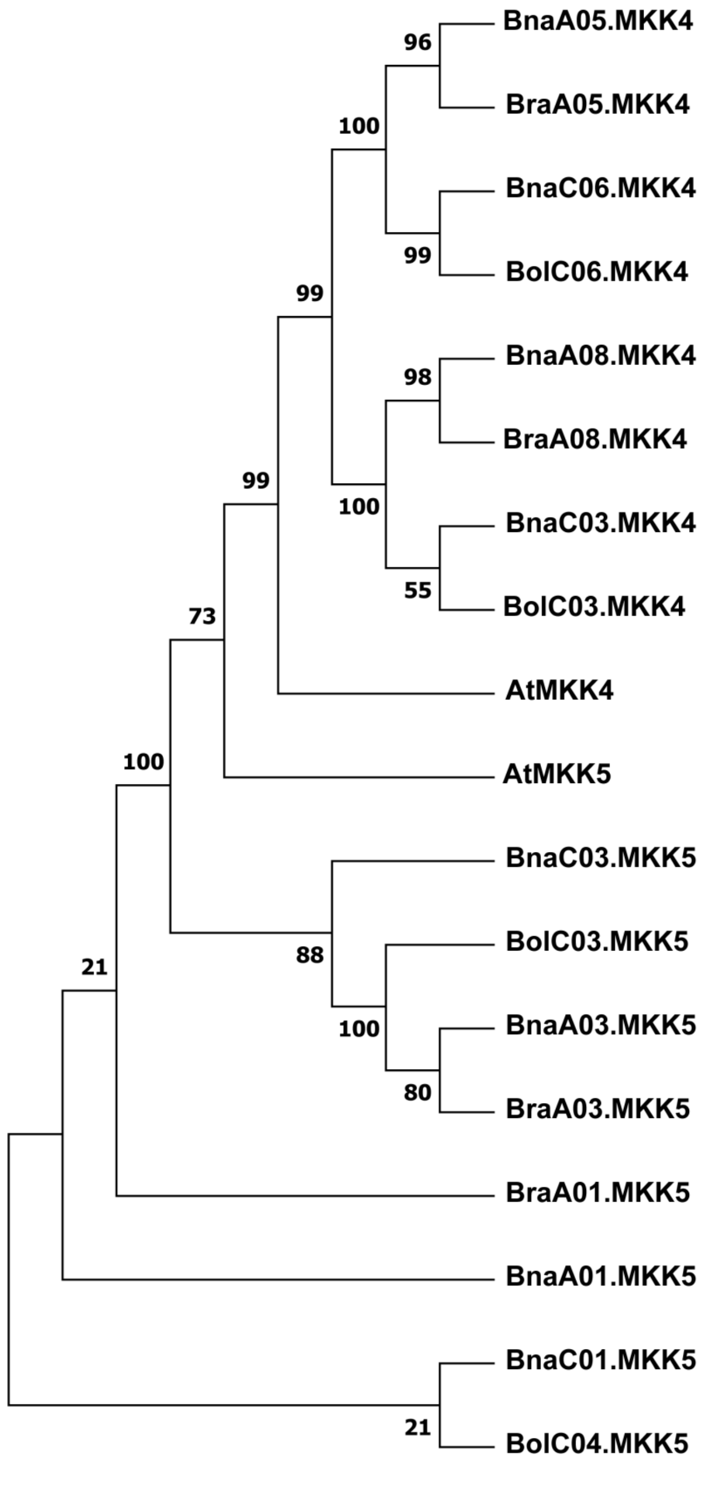

Figure S2: Phylogenetic analysis showing the relationship between each copy of B. napus MKK5 and MKK4/5 in *Brassica* species, as well as MKK4/5 in *Arabidopsis*.

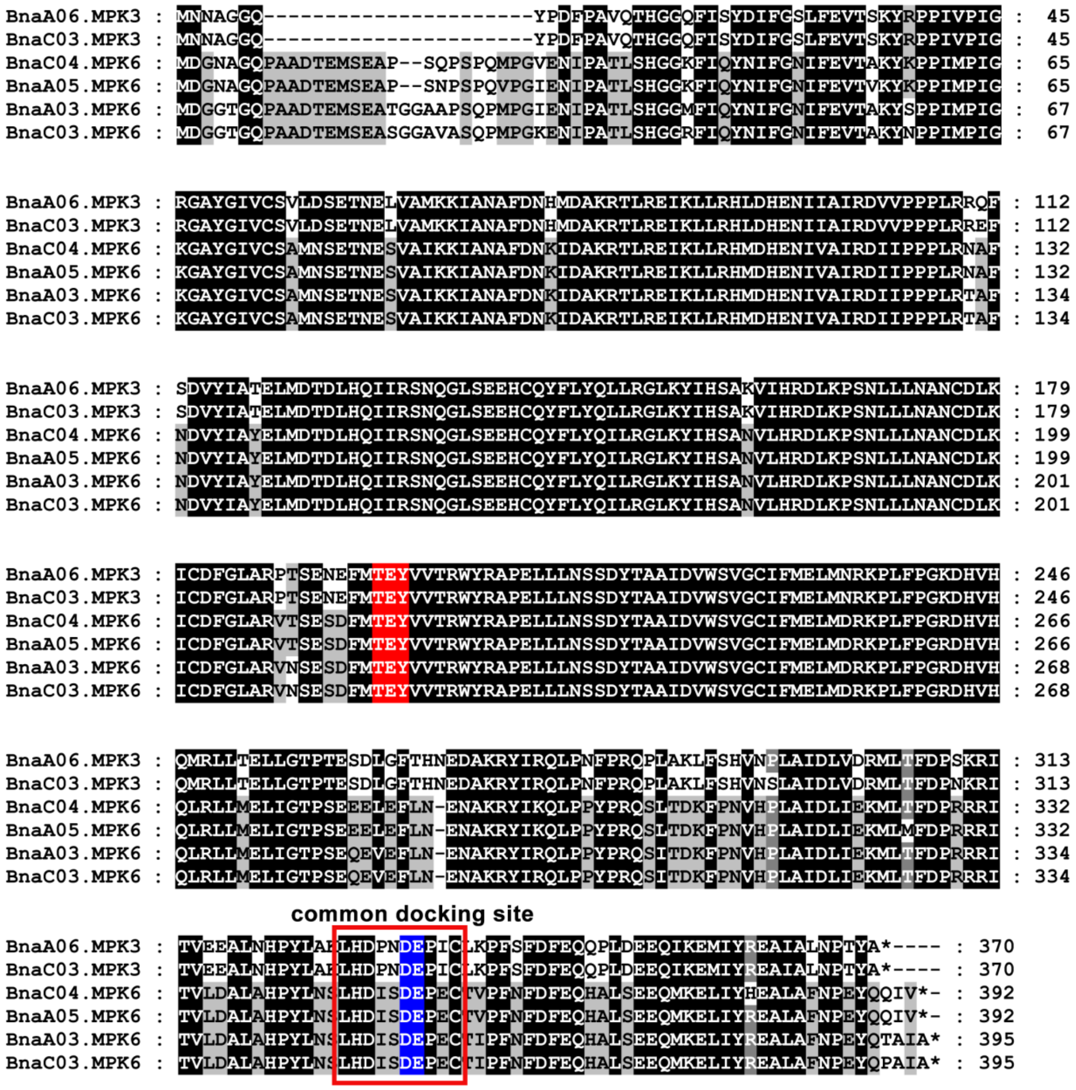

Figure S3: Sequence alignment of *B. napus* MPK3/6 copies. The identical amino acids and similar amino acids were displayed with black and grey shading respectively. TEY motif was marked with red; common docking site was marked with red square frame, and two adjacent acidic residues was highlighted in blue.
