# Supplementary material for: BnaA03.MKK5-BnaA06.MPK3/BnaC03.MPK3 Module Positively Contributes to Sclerotinia sclerotiorum Resistance in Brassica napus
Source: Plants (Basel). 2022 Feb 24;11(5):609. doi: 10.3390/plants11050609 (PMC8912397; doi:10.3390/plants11050609)
Supplement: Supplementary file 1 [file plants-11-00609-s001.zip › Table S1.pdf]

Table S1: Lesion areas of infected oilseed rape leaves after 48 h incubation with *S. Sclerotiorum*

A: Lesion areas of *BnaA03.MKK4* overexpression lines

**Sample ID**	**Lesion area(cm^2)**			**Mean value**	***P* value**
MKK4-OE #6	1.995	2.478	2.352	2.275	0.002375136
MKK4-OE #11	2.862	1.472	1.748	2.027	0.015821433
MKK4-OE #12	2.38	2.091	4.702	3.058	0.378975367
MKK4-OE #14	1.696	2.063	2.899	2.219	0.014063069
MKK4-OE #15	2.276	1.06	2.191	1.842	0.009126593
MKK4-OE #16	2.266	1.584	2.261	2.037	0.003196917
MKK4-OE #23	3.832	4.321	4.003	4.052	0.541635051
WT	4.076	3.521	4.088	3.895	

B: Lesion areas of *BnMPK3* transgenic lines

**Sample ID**	**Lesion area(cm^2)**			**Mean value**	***P* value**
MPK3-A6-OE #3	1.517	1.628	2.703	1.949	0.024422845
MPK3-A6-OE #4	1.95	2.122	2.626	2.233	0.008393751
MPK3-A6-OE #8	1.536	1.068	2.397	1.667	0.014486075
MPK3-C3-OE #16	1.7	2.475	2.239	2.138	0.008948213
MPK3-C3-OE #25	1.941	2.594	1.315	1.950	0.02277573
MPK3-C3-OE #30	1.54	2.167	2.287	1.998	0.006212795
MPK3-CR #44	4.054	4.22	4.696	4.323	0.010006986
MPK3-CR #49	4.194	4.7745	6.135	5.035	0.042974373
MPK3-CR #61	5.0265	4.275	4.683	4.662	0.005028396
WT	3.208	3.524	3.247	3.326	
